# Awareness and interest in IQOS heated tobacco products among youth in Canada, England & the United States.

**DOI:** 10.1136/tobaccocontrol-2018-054654

**Published:** 2019-01-29

**Authors:** Christine D. Czoli, Christine M. White, Jessica L. Reid, Richard J. O’Connor, David Hammond

**Affiliations:** 1 School of Epidemiology and Public Health, University of Ottawa, Ottawa, Canada; 2 Heart and Stroke Foundation of Canada, Ottawa, Canada; 3 School of Public Health and Health Systems, University of Waterloo, Waterloo, Canada; 4 Department of Health Behavior, Roswell Park Comprehensive Cancer Centre, Buffalo, USA

## Abstract

**Introduction:**

Heated tobacco products (HTPs), such as IQOS, have been introduced in a growing number of international markets. However, little is known about perceptions of HTP products among youth.

**Methods:**

Data are from Wave 1 of the International Tobacco Control Youth Tobacco and E-cigarette Survey (2017), a web-based cohort survey of 16- to 19-year-olds from Canada, England, and the United States (US). Respondents (n=12,064) were shown an image of IQOS and asked about their awareness, interest in trying, and susceptibility to trying the product. Youth awareness, interest in trying, and susceptibility to trying IQOS were analyzed using descriptive statistics, and logistic regression models were used to examine correlates of these outcomes.

**Results:**

Overall, 7.0% of youth reported awareness of IQOS (England=5.6%, Canada=6.4%, and US=9.1%) and 38.6% expressed interest in trying the product (England=41.8%, Canada=33.0%, and US=40.9%). Within each country, all key outcomes varied by smoking status: greater proportions of youth who were currently smoking or had a history of smoking reported being aware of, interested in trying, and susceptible to trying IQOS. Interest and susceptibility to trying IQOS were associated with male sex, current tobacco use, and current e-cigarette use. Across all countries, susceptibility to trying IQOS (25.1%) was higher than for tobacco cigarettes (19.3%), but lower than for e-cigarettes (29.1%).

**Conclusions:**

Awareness of heated tobacco products, such as IQOS, is emerging among youth in Canada, England, and the US. Interest in trying these products is very high among smokers, but also present among non-smokers.

## INTRODUCTION

Over the last decade, the nicotine market has rapidly evolved with the emergence of e-cigarettes. More recently, a new generation of vapourized products has emerged, which are often referred to as ‘heated’ tobacco sticks or ‘heated tobacco products’ (HTPs). Like e-cigarettes, these products use heat to volatilize nicotine below the point of combustion, so that consumers inhale an aerosol rather than smoke.[1] Unlike e-cigarettes, which heat nicotine from a liquid solution, HTPs heat cigarette-like tobacco ‘sticks’. The constituents of the tobacco sticks that are heated and the type of heating device can vary across brands and product platforms.[1] In essence, HTPs occupy a position in the nicotine spectrum between conventional ‘smoked’ cigarettes and e-cigarettes: their tobacco substrate resembles that of regular cigarettes, while their mechanism of delivering nicotine primarily through aerosol resembles that of e-cigarettes.

HTPs are marketed as reduced risk tobacco products,[2] similar to previous generations of products such as Eclipse, Advance, Omni, and Accord.[3,4] The central principle underlying this marketing is that HTPs are likely to be less harmful than tobacco cigarettes because they do not combust tobacco, and therefore reduce exposure to the many harmful constituents of tobacco smoke.[2,5] Most research conducted by tobacco manufacturers is consistent with this premise, showing that HTP aerosol contains lower levels of toxic emissions compared to cigarette smoke, and that use of HTPs is associated with reductions in biomarkers of exposure to several harmful and potentially harmful tobacco smoke constituents.[6–8] However, independent examination of this evidence has revealed significant methodological issues with these findings,[2] raising concerns regarding associations between HTP aerosol exposure and impaired vascular endothelial function,[9] pulmonary effects,[10] and liver toxicity.[11] Evidence from independent research examining HTPs is limited. A modelling analysis quantifying the harms of vapourized nicotine products relative to tobacco smoke distinguished these products along a spectrum spanning five orders of magnitude, with the greatest risks posed by tobacco cigarettes, followed sequentially by HTPs, e-cigarettes, and nicotine inhalers.[12] In an *in vitro* study using human bronchial epithelial cells, HTP aerosol resulted in significantly higher cytotoxicity than e-cigarette aerosol, but less than that of cigarette smoke.[13] However, product testing of HTP aerosols have produced mixed findings regarding the levels of harmful and potentially harmful constituents in HTP aerosol relative to tobacco smoke.[14–17] Further research is required to understand the potential health impacts of these novel products at the individual and population levels.

HTPs such as IQOS, Ploom, and Glo are being sold in an increasing number of countries, and are marketed as premium products for tech-savvy users.[18] Considerable market growth has been documented in Japan, where HTPs have been available since 2014.[15] In a 2015 national survey of Japanese adults, 48% were aware of e-cigarettes and HTPs, with 7% reporting having ever used these products, and 1.3% reporting use in the last 30 days.[19] A follow-up survey conducted two years later (2017) indicated that use of IQOS in the last 30 days had increased to 3.6%, while rates of use of other HTPs had remained low.[20] Notably, the Japanese market is distinct in that nicotine-containing e-cigarettes are not permitted for sale,[19] and the rise of HTPs has been accompanied by a decline in tobacco cigarette sales.[21] To date, consumer uptake of HTPS has been limited in other markets. In Canada, Glo (British American Tobacco; BAT) and IQOS (Philip Morris International; PMI) are available in select cities and provinces.[22,23] Because HTPs represent a ‘new’ product category, they do not fall into any pre-existing regulatory classification. In Canada, HTPs are regulated as tobacco products,[24] although they are not required to carry health warnings. In England, HTPs have been available since 2014,[25] and are regulated as ‘other smoking tobacco and chewing tobacco’ products. Limited evidence suggests that consumer awareness and use of HTPs among adults is low.[26]

IQOS, Glo and other modern HTPs are not available for sale in the US without FDA approval as a new tobacco product. In May 2017, PMI submitted an application to permit the sale of IQOS in the US, along with a modified risk tobacco product (MRTP) application for its IQOS system and three types of its ‘HeatStick’ products (https://www.fda.gov/tobaccoproducts/labeling/marketingandadvertising/ucm546281.htm). In January 2018, the US Food and Drug Administration’s (FDA) Tobacco Products Scientific Advisory Committee (TPSAC) reviewed the evidence submitted by PMI and concluded that, although the product reduces exposure to harmful constituents found in tobacco,[26] PMI had not provided adequate evidence to demonstrate that such reduced exposure would likely translate into measurable reductions in tobacco-related diseases.[27] As part of the decision, the Committee expressed a range of opinions regarding the likelihood that youth never-smokers would become established users of the IQOS system, and noted the absence of data among youth.[27] Indeed, virtually all of the existing evidence on use of HTPs has focused on adults and established tobacco users. Although surveys of Japanese consumers indicate greater rates of use among younger individuals,[20] to our knowledge, there is little or no data on use or perceptions among youth, including youth smokers and non-smokers. While TPSAC recommended denial of the MRTP application, the final FDA decision is pending.

The current study sought to fill this gap by examining awareness and interest in IQOS among youth in Canada, England, and the US. The study also compared susceptibility to IQOS with susceptibility to e-cigarettes and conventional cigarettes, and examined differences among sub-groups.

## METHODS

### Data source

Data are from Wave 1 of the International Tobacco Control Policy Evaluation Project (ITC) Youth Tobacco and E-cigarette Survey, conducted in Canada, England, and the United States. Data were collected via self-completed web-based surveys conducted in July/August 2017 with youth aged 16 through 19. Respondents were recruited through Nielsen Consumer Insights Global Panel and their partners’ panels, either directly or through their parents. Email invitations (with a unique link) were sent to a random sample of panelists (after targeting for age criteria); panelists known to be ineligible were not invited. A restriction on small screen size was applied to ensure that images presented in the survey could be viewed with a minimum amount of scrolling. Thus, panelists who were not between the ages of 16 and 19 and/or had no children between the ages of 16 and 19, and/or reported use of a mobile device while completing the survey were deemed ineligible. The survey was conducted in English in all countries, as well as French in Canada, and took approximately 15 minutes to complete. The same survey measures were used in all countries, with the exception of race/ethnicity, region, and education questions, which were based on census questions in each country.

Respondents provided consent prior to completing the survey. In total, 379,777 invitations were sent to panelists (192,736 directly to youth and 187,041 to parents), and 34,470 potential respondents accessed the survey link for a participation rate of 9.1%.[28] As a data integrity check, respondents were asked to select the current month from a list. The month selected by respondents was compared to the month when the survey was submitted. Respondents with a month discrepancy were excluded from the analysis, unless the selected month was within two days of the date the survey was submitted. Respondents received remuneration in accordance with their panel’s usual incentive structure. The study was reviewed by and received ethics clearance through a University of Waterloo Research Ethics Committee (ORE#21847) and the King’s College London Psychiatry, Nursing & Midwifery Research Ethics Subcommittee. A full description of the study methods can be found in the Technical Report.[29]

### Measures

**Figure 1:**
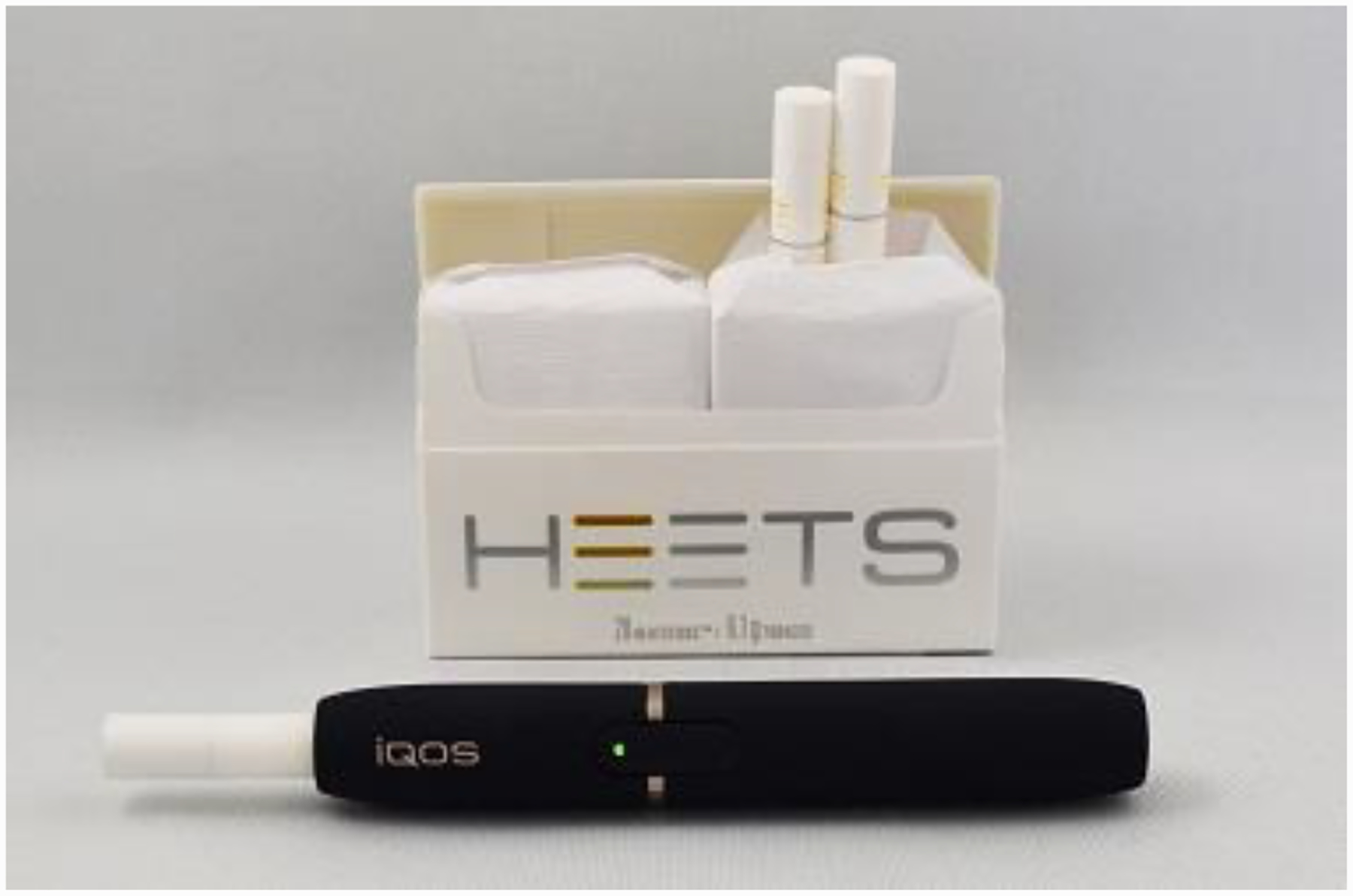
IQOS heated tobacco product device and tobacco sticks

Respondents were shown an image of the IQOS product (see Figure 1) and asked several questions. Respondents were asked about their awareness of the product, with the question: “*Have you heard of a product called IQOS, which heats a stick of tobacco instead of burning it?*”, with response options ‘yes’ or ‘no’. Next, respondents were asked about their interest in trying the product, with the question: “*Would you be interested in trying this product?*”, with response options ‘definitely not’, ‘probably not’, ‘probably yes’, and ‘definitely yes’. Respondents were also asked a measure of susceptibility established for tobacco cigarettes (“*If one of your best friends were to offer you a cigarette, would you smoke it?*”), which was adapted for IQOS (“*If one of your best friends were to offer you this product, would you try it?*”) and for e-cigarettes (“*If one of your best friends were to offer you an e-cigarette/vaping device, would you use it?*”), and included the response options ‘definitely not’, ‘probably not’, ‘probably yes’, and ‘definitely yes’. Respondents could also select the response options ‘don’t know’ or ‘refuse to answer’ for all questions.

Smoking status was defined using the following categories: *never smokers* had never smoked a cigarette; *experimental smokers* had smoked less than 100 cigarettes in their lifetime; *former smokers* had smoked 100 cigarettes in their lifetime, but did not report smoking in the past 30 days; and *current smokers* had smoked 100 cigarettes in their lifetime and reported smoking in the past 30 days. Vaping status was defined using parallel categories, including the requirement for *former vapers* and *current vapers* to have vaped on 100 days in their lifetime.

### Analysis

Sample weights were constructed using a raking algorithm. First, respondents were divided into three broad cigarette smoking categories: never smokers, experimental smokers (smoked <100 cigarettes lifetime), and current/former smokers (smoked >100 cigarettes lifetime). Raking was then performed based on geographic region (state/province/region), language in Canada (English or French), and the following cross-classifications: sex by smoking, age (16–17 or 18–19) by smoking, and age by race/ethnicity in the US (white/Caucasian, African-American, or other). Finally, weights were rescaled to sample size within each country/condition, to allow for comparisons between countries with different population sizes. Estimates reported are weighted unless otherwise specified.

Differences in key outcomes across countries were examined using chi-square tests. Logistic regression models were estimated to examine differences in outcomes between countries, adjusting for age, sex, smoking status, and vaping status. Differential trends by country were tested by examining two-way interactions of country with age, sex, smoking status, and vaping status. Outcomes were modelled as binary variables: awareness of IQOS (‘no’ vs. yes’); interest in trying IQOS (‘definitely not’ vs. any other response); and susceptibility to trying IQOS (‘definitely not’ vs. any other response). Susceptibility to trying conventional cigarettes, IQOS, and e-cigarettes were examined among subsamples of youth *never smokers and never vapers*, adapting the conventional approach by Pierce et al.[30] Analyses were conducted using IBM SPSS Statistics v.24.

## RESULTS

After excluding those who failed the data quality check (n=382) and those missing any of the variables used in the weighting (n=1,022), 12,064 respondents comprised the analytic sample. Characteristics of the sample, unweighted and weighted, are shown in Table 1.

Key binary outcomes are presented by country and smoking status in Table 2 (for the presentation of outcomes with the full range of responses, see Supplementary Table 1). Youth in the US reported the greatest levels of awareness of IQOS (χ^2^_(df=2, n=11,770)_=41.3, p<0.001), while Canadian youth reported the least interest and susceptibility to trying IQOS (χ^2^_(df=2, n=11,416)_=75.8, p<0.001) and (χ^2^_(df=2, n=11,499)_=98.4, p<0.001), respectively. As shown in Table 2, key outcomes varied by smoking status within each country: although awareness, interest and susceptibility to trying IQOS were reported by a greater proportion of youth who were currently smoking or had a history of smoking, considerable proportions of never smokers in each country also reported being interested in and susceptible to trying this novel product.

**Figure 2:**
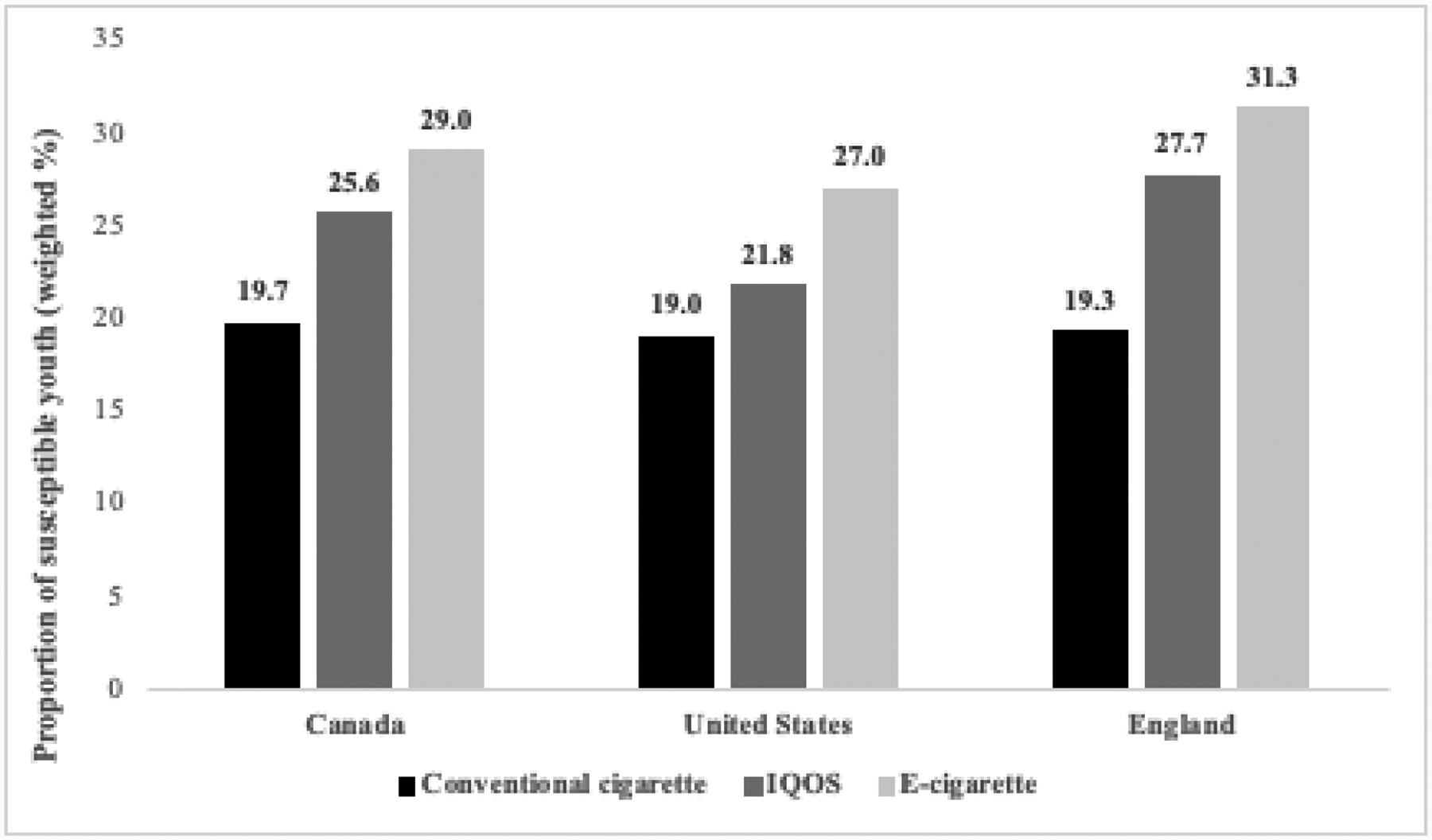
Susceptibility to trying conventional cigarettes, IQOS, and e-cigarettes among never-smoking and never-vaping youth, by country (N=7,012) Note: Analyses conducted using weighted data. Respondents with missing data are not included in weighted estimates.

Among youth *never smokers and never vapers* across all countries, susceptibility to trying IQOS (25.1%) was higher than for tobacco cigarettes (19.3%), but lower than for e-cigarettes (29.1%). This pattern across products was also reflected within each country (see Figure 2).

Table 3 presents results of separate multivariate logistic regression analyses for IQOS awareness, interest in trying, and susceptibility to trying among youth, across countries. Compared to Canada, youth in the US were significantly more likely to be aware of IQOS, and significantly less susceptible to trying the product; in contrast, youth in England were significantly less likely to be aware of IQOS, and significantly more susceptible to trying the product.

Across all countries, males were significantly more likely to report IQOS awareness, interest in trying, and susceptibility to trying. Several differences in youth interest in trying IQOS were noted by country and sex: interest in trying the product was more likely to be reported by females in England compared to those in Canada (aOR=1.36 (95% CI 1.08, 1.70), p=0.008), and by males in the US compared to England (aOR=1.35 (95% CI 1.08, 1.69), p=0.009). With respect to age, awareness of IQOS decreased significantly with increasing age in Canada and England, while in the US, older youth were significantly more likely to report awareness of the product (vs. Canada: aOR=1.25 (95% CI 1.06, 1.48), p=0.008; vs. England: aOR=1.23 (95% CI 1.03, 1.46), p=0.021). Similarly, older youth in the US were more likely to report interest in trying IQOS compared to those in Canada and England (aOR=1.12 (95% CI 1.01, 1.25), p=0.032, and aOR=1.17 (95% CI 1.05, 1.30), p=0.005, respectively). Susceptibility to trying IQOS decreased significantly with increasing age among youth in all countries.

Smoking status was significantly associated with all IQOS outcomes. Overall, compared to *never smokers*, *experimental smokers* and *former smokers* were significantly more likely to be aware of the product. Trends differed by country, with *current smokers* in the US significantly more likely to be aware of IQOS than those in Canada and England (aOR=2.10 (95% CI 1.14, 3.86), p=0.018, and aOR=2.75 (95% CI 1.46, 5.17), p=0.002, respectively). In addition, *experimental smokers* in Canada were significantly more likely to report awareness of IQOS than those in England (aOR=1.92 (95% CI 1.11, 3.33), p=0.019). Across all countries, *experimental smokers, former smokers*, and *current smokers* were all significantly more likely to report interest in trying IQOS and susceptibility to trying IQOS. However, *former smokers* in Canada were significantly less likely than those in England and the US to report interest in trying IQOS (aOR=0.28 (95% CI 0.12, 0.65), p=0.003, and aOR=0.31 (95% CI 0.12, 0.80), p=0.016), respectively). Compared to Canada, *current smokers* in the US were significantly more likely to report being interested in trying IQOS (aOR=2.18 (95% CI 1.08, 4.40), p=0.031). Susceptibility to trying IQOS also differed by country and smoking status, with *former smokers* in Canada significantly less susceptible to trying IQOS compared to those in England and the US (aOR=0.20 (95% CI 0.08, 0.51), p=0.001, and aOR=0.33 (95% CI 0.13, 0.83), p=0.019, respectively).

With respect to use of e-cigarettes, compared to *never vapers*, *experimental vapers, former vapers*, and *current vapers* were all significantly more likely to be aware of, interested in trying, and susceptible to trying IQOS across all countries. In addition, *former vapers* in Canada were significantly more likely than those in the US to report awareness of IQOS (aOR=6.68 (95% CI 1.55, 28.86), p=0.011), as were *current vapers* in England compared to those in the US (aOR=4.02 (95% CI 1.66, 9.77), p=0.002).

## DISCUSSION

The study findings indicate that awareness of IQOS among youth is emerging in Canada, England, and the United States. In particular, levels of awareness were higher among males, and among youth who used tobacco cigarettes or e-cigarettes. Both Canada and England have comprehensive restrictions on advertising and promotion of tobacco products, which have likely had an impact on the industry’s ability to promote awareness of these products. Indeed, PMI has been promoting IQOS on packages of conventional cigarettes as one of the only marketing channels available.[31] In addition, the Canadian market has largely been restricted to several large urban cities, which may impact consumer awareness of IQOS.[22,23]

Interest and susceptibility to trying IQOS among youth were also associated with male sex, use of tobacco cigarettes, and use of e-cigarettes. However, the magnitude of associations between tobacco use and IQOS outcomes was markedly greater than those for e-cigarette use, underscoring the predominance and consistency of cigarette smoking as a factor associated with use of other tobacco products.[32] Nevertheless, some youth with no history of tobacco or e-cigarette use also expressed interest and susceptibility to trying IQOS: in all countries, approximately one-fifth of never-users expressed interest in trying IQOS, while approximately one-third were considered susceptible to trying the product. Although it is unclear whether and how IQOS may affect rates of smoking, these results raise concerns as to the broad appeal of these products among youth, particularly among those with no past use of tobacco or nicotine products.

Across and within all countries, susceptibility to trying IQOS was greater than for tobacco cigarettes, but lower than for e-cigarettes. An FDA review of consumer studies submitted by PMI concluded that some youth non-smokers would be expected to experiment with IQOS, but speculated that interest may be lower than for e-cigarettes due to predominantly ‘negative’ associations with tobacco among young non-smokers.[6] The current findings appear to be consistent with this suggestion. More generally, the findings reflect the evolving tobacco/nicotine market, in which novel and alternative products are playing an increasingly important role.[32] Understanding youth susceptibility and perceptions of these various products may help inform youth substance use prevention. Qualitative evidence suggests that IQOS packaging and marketing may have particular appeal among youth and young adults, given the important role that technology plays in their lives.[33] Future research examining whether youth view HTPs as appealing or harmful relative to tobacco cigarettes and other products is critically important to understand the potential role of these products in the rapidly evolving market. It is also worth noting that the current study tested interest and susceptibility for an ‘unflavoured’ version of the HTP tobacco sticks. However, the MRTP applications to the US FDA and tobacco sticks on the market in different countries include varieties with menthol flavours (e.g., Marlboro Smooth Menthol and Marlboro Fresh Menthol) which are associated with greater appeal among youth and young adults.[34,35] Therefore, actual levels of interest and susceptibility among youth may be higher than estimates from the current study.

Although some differences in levels of awareness, interest in trying, and susceptibility to trying IQOS were found between countries, these differences were not consistent, likely reflecting the novelty of HTPs. Continued monitoring of awareness, interest in trying and prevalence of use of HTPs is needed to better understand the potential public health impact of these products, particularly across jurisdictions with differing policies for various tobacco/nicotine products as well as differing cultural values, which appear to play a role in how these products are perceived.[33] Robust and independent research will be particularly important, given PMI’s pledge to develop and market reduced-risk alternatives to cigarette smoking.[36]

### Strengths and limitations

To our knowledge, the current study is the first to examine youth awareness and interest in trying HTPs in national samples across several countries. However, the study has several limitations. The proliferation of new tobacco products presents a challenge to the traditional product definitions used in population surveys. In order to assist respondents, an image of IQOS and the ‘HeatStick’ products were displayed on screen; nevertheless, it is possible that some respondents confused the product either with a conventional cigarette or an e-cigarette. This limitation may be reflected in relatively high levels of IQOS awareness among youth in the US, despite the fact that it is not marketed in the US; alternatively, youth in the US may be aware of these products via the internet, despite its absence on the US market. In addition, the current study only assessed awareness of interest in a single HTP. Although IQOS appears to be the most prominent of the new generation of HTPs, others have also been introduced in Canada and England, including BAT’s Glo. Study samples were recruited using national online commercial panels, but were not probability-based. However, the sample was weighted by sex, age, region and smoking status, and estimates from the study sample were very similar to national benchmark surveys in each country (Canada: Canadian Tobacco, Alcohol and Drugs Survey; England: Opinions and Lifestyle Survey; US: Monitoring the Future survey, and National Youth Tobacco Survey).[29] For example, the prevalence of vaping and cannabis use in the study sample are typically within 1–3 percentage points of national estimates in all three countries. Finally, the study used a cross-sectional design, which does not allow for causal inferences to be drawn between the examined IQOS outcomes and various correlates. Longitudinal research is needed to evaluate the temporal nature of these associations, as well as product uptake and use.

Youth awareness of heated tobacco products is emerging in Canada, England, and the United States. Interest in trying these products is very high among smokers, but also present among non-smokers. As the US FDA’s TPSAC report noted, the public health impact of HTPs, such as IQOS, depends not only on whether they are less harmful than conventional cigarettes, but whether they help to increase or decrease the prevalence of smoking.[27] The extent to which these products appeal to youth represents a fundamentally important component of this equation, particularly if HTPs have levels of appeal closer to conventional cigarettes than most other non-combustible forms of nicotine.

## Supplementary Material

Supplementary Table 1

## Figures and Tables

**Table 1:** Sample characteristics, by country (N=12,064)

Characteristic	Unweighted (N=12,064)						Weighted (N=12,064)					
	Canada (n=4,008)		United States (n=4,086)		England (n=3,970)		Canada (n=4,008)		United States (n=4,086)		England (n=3,970)	
	% (n)						% (n)					
**Age**												
Mean (SD)	17.7 (1.0)		17.6 (1.1)		17.7 (1.0)		17.6 (1.0)		17.5 (1.1)		17.5 (1.0)	
**Sex**												
Male	34.7	(1391)	39.6	(1619)	42.7	(1697)	51.6	(2068)	53.3	(2178)	55.3	(2195)
Female	65.3	(2617)	60.4	(2467)	57.3	(2273)	48.4	(1940)	46.7	(1908)	44.7	(1775)
**Smoking status**												
Never smoker	70.6	(2829)	67.6	(2763)	59.2	(2349)	78.4	(3143)	60.7	(2481)	61.9	(2459)
Experimental smoker	24.7	(989)	27.4	(1118)	33.7	(1337)	8.5	(341)	28.0	(1143)	20.6	(817)
Former smoker	0.6	(23)	0.6	(26)	0.8	(32)	1.6	(63)	1.3	(55)	1.8	(73)
Current smoker	4.2	(167)	4.4	(179)	6.3	(252)	11.5	(462)	10.0	(408)	15.6	(621)
**Vaping status**												
Never vaper	72.8	(2899)	68.1	(2761)	67.2	(2643)	74.4	(2958)	64.3	(2607)	65.7	(2567)
Experimental vaper	25.2	(1002)	29.0	(1175)	31.1	(1222)	22.4	(890)	30.5	(1239)	30.9	(1209)
Former vaper	0.4	(17)	0.7	(28)	0.5	(18)	0.7	(28)	1.0	(42)	0.8	(33)
Current vaper	1.6	(62)	2.2	(90)	1.2	(49)	2.5	(101)	4.1	(168)	2.6	(101)

Note: Respondents with missing data for vaping status are not included in totals: unweighted sample n=98 (Canada=28; US=32; England=38); weighted sample n=121 (Canada=31; US=30; England=60).

**Table 2:** Prevalence estimates of IQOS awareness, interest in trying, and susceptibility to trying among youth, by country and smoking status (N=12,064)

	Aware of IQOS		Interested in trying IQOS		Susceptible to trying IQOS	
	Weighted % (n)					
**Canada (n=4,008)**	**6.4**	**(248)**	**33.0**	**(1253)**	**40.1**	**(1537)**
Never smoker (n=3143)	5.0	(152)	22.8	(702)	28.8	(881)
Experimental smoker (n=341)	14.2	(47)	61.3	(190)	72.6	(227)
Former smoker (n=63)	18.4	(12)	52.7	(30)	49.5	(27)
Current smoker (n=462)	8.5	(38)	92.1	(332)	98.6	(402)
**United States (n=4,086)**	**9.1**	**(363)**	**40.9**	**(1586)**	**46.1**	**(1783)**
Never smoker (n=2481)	5.9	(142)	21.4	(519)	25.9	(620)
Experimental smoker (n=1143)	14.1	(158)	64.9	(673)	72.0	(749)
Former smoker (n=55)	5.9	(3)	80.3	(39)	75.8	(38)
Current smoker (n=408)	15.3	(59)	96.3	(355)	97.6	(377)
**England (n=3,970)**	**5.6**	**(217)**	**41.8**	**(1564)**	**51.4**	**(1951)**
Never smoker (n=2459)	4.1	(99)	23.2	(556)	31.2	(745)
Experimental smoker (n=817)	8.8	(70)	63.1	(471)	77.1	(585)
Former smoker (n=73)	13.0	(9)	79.4	(54)	85.3	(61)
Current smoker (n=621)	6.3	(39)	91.4	(483)	97.6	(560)

Note: Analyses conducted using weighted data. Respondents with missing data are not included in weighted estimates.

**Table 3:** Estimates from separate logistic regression models of IQOS awareness, interest in trying, and susceptibility to trying among youth in Canada, England, and the United States

Variable	Aware of IQOS (n=11,704)	Interested in trying IQOS (n=11,396)	Susceptible to trying IQOS (n=11,424)
	aOR^a^ (95% CI)		
**Age**	0.90** (0.84, 0.96)	0.98 (0.94, 1.03)	0.95* (0.91, 0.99)
**Sex**			
Female	Ref	Ref	Ref
Male	1.35*** (1.16, 1.57)	1.21*** (1.10, 1.32)	1.21*** (1.10, 1.32)
**Country**			
Canada	Ref	Ref	Ref
United States	1.20* (1.01, 1.44)	1.00 (0.90, 1.13)	0.87* (0.78, 0.97)
England	0.71** (0.59, 0.87)	1.03 (0.92, 1.16)	1.17** (1.05, 1.30)
**Smoking status**			
Never smoker	Ref	Ref	Ref
Experimental smoker	1.85*** (1.53, 2.25)	3.86*** (3.43, 4.34)	4.39*** (3.89, 4.96)
Former smoker	1.96** (1.23, 3.14)	4.02*** (2.81, 5.75)	2.47*** (1.72, 3.56)
Current smoker	1.24 (0.96, 1.59)	23.96*** (18.79, 30.56)	52.68*** (35.84, 77.44)
**Vaping status**			
Never vaper	Ref	Ref	Ref
Experimental vaper	1.96*** (1.63, 2.35)	2.75*** (2.46, 3.06)	3.28*** (2.93, 3.67)
Former vaper	2.06* (1.10, 3.85)	2.18** (1.26, 3.79)	3.59*** (1.84, 7.02)
Current vaper	2.32*** (1.61, 3.33)	3.71*** (2.49, 5.53)	3.91*** (2.49, 6.12)

Abbreviations: aOR=Adjusted odds ratio; CI=Confidence interval; Ref=Reference category.

Note: Analyses conducted using weighted data.

* p<0.05,

** p<0.01,

*** p<0.001

a Odds ratios adjusted for age, sex, country, smoking status, and vaping status.

## What this paper adds

- To date, there is little or no data on perceptions or use of heated tobacco products (HTPs) among youth, including youth smokers and non-smokers.
- Findings from the current study show that youth awareness of HTPs is emerging in Western countries. Interest in trying these products is very high among smokers, but also present among non-smokers.
- The findings are directly relevant to policy, given that youth appeal of HTPs represents a fundamental component of evaluating their public health impact.
