# Supplementary material for: Awareness and interest in IQOS heated tobacco products among youth in Canada, England & the United States
Source: Tob Control. Author manuscript; Available in PMC 2021 Mar 15. (PMC7958490; doi:10.1136/tobaccocontrol-2018-054654)
Supplement: Supplementary Table 1 [file NIHMS1674357-supplement-Supplementary_Table_1.docx]

**Supplementary Table 1:** **Prevalence estimates of IQOS awareness, interest in trying, and susceptibility to trying among youth, by country (N=12,064)**

| **Measure** | | **Canada**  **(n=4,008)** | | | **United States**  **(n=4,086)** | | | **England**  **(n=3,970)** | | |
| --- | --- | --- | --- | --- | --- | --- | --- | --- | --- | --- |
|  | | **Weighted % (n)** | | | | | | | | |
| **Awareness:** *Have you heard of a product called IQOS, which heats a stick of tobacco instead of burning it?* | | | | | | | | | | |
|  | No | 93.6 | (3641) | | 90.9 | (3625) | | 94.4 | (3676) | |
|  | Yes | 6.4 | (248) | | 9.1 | (363) | | 5.6 | (217) | |
| **Interest in trying:** *Would you be interested in trying this product?* | | | | | | | | | | |
|  | Definitely not | 67.0 | | (2547) | 59.1 | | (2288) | 58.2 | | (2178) |
|  | Probably not | 21.9 | | (834) | 25.1 | | (974) | 25.8 | | (967) |
|  | Probably yes | 8.1 | | (310) | 11.7 | | (452) | 12.7 | | (475) |
|  | Definitely yes | 2.9 | | (110) | 4.1 | | (160) | 3.2 | | (121) |
| **Susceptibility:** *If one of your best friends were to offer you this product, would you try it?* | | | | | | | | | | |
|  | Definitely not | 59.9 | | (2296) | 53.9 | | (2088) | 48.6 | | (1844) |
|  | Probably not | 22.9 | | (876) | 23.1 | | (893) | 24.8 | | (942) |
|  | Probably yes | 12.5 | | (481) | 15.4 | | (595) | 20.0 | | (760) |
|  | Definitely yes | 4.7 | | (180) | 7.6 | | (296) | 6.5 | | (248) |

Note: Analyses conducted using weighted data.

Respondents with missing data are not included in weighted totals: awareness of IQOS n=295 (Canada=119; US=98; England=77); interest in trying IQOS n=649 (Canada=208; US=213; England=228); susceptibility to trying IQOS n=564 (Canada=175; US=214; England=175).
